# Thermal shock resistance of additive manufactured Inconel 718 by concentrated solar energy

**DOI:** 10.1038/s41598-025-92332-x

**Published:** 2025-03-04

**Authors:** Juan de Damborenea, Ana Conde, Gloria Rodriguez-Donoso, Fernando Agulló-Rueda, Maria Angeles Arenas

**Affiliations:** 1 Surface Engineering, Corrosion and Durability Department, National Center for Metallurgical Research (CENIM-CSIC), Avda. Gregorio del Amo 8, 28040 Madrid, Spain; 2 E.T.S Ingeniería Industrial, Universidad de Castilla-La Mancha (ETSII-UCLM), Avda. Camilo José Cela S/N, 13071 Ciudad Real, Spain; 3 Instituto de Ciencia de Materiales de Madrid, (ICMM), CSIC, Sor Juana Inés de la Cruz 3, 28049 Madrid, Spain

**Keywords:** Thermal shock, Concentrated solar power, Inconel 718, Oxidation, Additive manufacturing, Materials science, Structural materials, Metals and alloys

## Abstract

Concentrated Solar Power (CSP) is a powerful tool for simulating the extreme high-temperature conditions that metallic materials encounter. Using a vertical parabolic solar furnace, it was possible to perform heating and cooling cycles between 250 and 950 °C in approximately 250 s per cycle. This capability is particularly relevant for the development of solar receivers used in solar thermal plants. Additive Manufacturing (AM) offers the potential to create new compositions and geometries that can enhance the efficiency of these solar receivers. In this study, Ni-base superalloys—identified as suitable materials for high-temperature solar receivers—were produced using AM and tested in two conditions: as-built and after thermal treatment. These were compared with a forged reference alloy. The results revealed the formation of a protective oxide layer on the surface in all cases. However, the oxide layer on the samples fabricated by additive manufacturing appeared to be more compact and adherent compared to that formed on the reference alloy.

## Introduction

Photovoltaic solar energy (SPV) is widely available and environmentally friendly. However, its use in large-scale electricity production has only recently begun extended. According to the International Energy Agency^1^, global SPV capacity increased from 1.2TW in 2022 to 1.6TW in 2023, with new installations rising from 407.3GW to 446GW. This represents around 5.6% of total global electricity generation in 2023, up from 4.6% a year earlier. These figures are far from the electricity generated using Concentrated Solar Power (CSP), which has remained more or less stable at around 13.6 terawatt-hours in recent years^1^. Therefore, contrary to initial expectations, the utilisation rate remains stable at a low level, but with great potential for the future^2^. This has resulted in a continued lag between the actual performance and the projected SPV figures.

Concentrating solar power has been used for a long time. The best known and, also controversial application of concentrated solar power dates back to the famous story of Archimedes’ use of solar energy in the siege of Syracuse (213–212 BCE)^3,4^. Since then, the deployment of concentrated solar power has been unpredictable, with varying levels of success in terms of expectations and applications^5^. However, in last decade, the power plant construction seems to be revitalized, showing an interesting growth rate. Alami et al.^6^ recently reviewed the state of the art of CSP technologies. They found that the most commonly used technologies are parabolic trough, solar thermal tower, and linear Fresnel solar collectors. Currently, there are 114 fully operational CSP plants worldwide as well as 18 under construction.

Concentrating solar power exhibits considerable growth potential on a global scale, largely due to its substantial capacity to store thermal energy and deploy it when solar irradiation is no longer available. Trieb et al.^7^ estimated that globally, CSP could produce 3,000,000 TWh/year, which is several orders of magnitude higher than the global electricity consumption. In addition to the generation of electricity, concentrating solar power is capable of processing or synthesising materials, a feature that is attracting increasing interest in its development^8^. Furthermore, solar concentrating systems offer unique opportunities for high-temperature testing, and are cost-effective in comparison to conventional facilities.

CSP plants convert solar energy into electricity by focusing sunlight onto a receiver, which heats a high-temperature fluid that runs a turbine or engine. Power plants ranging from kW to MW can operate with ease. The efficiency of the process increases at higher temperatures, while cost-effectiveness and safety operation is maintained^9^. A complete description of the advances in central receivers can be found elsewhere^10^. As solar receivers operate under extreme conditions, materials that withstand high temperatures, are corrosion-resistant, have high thermal conductivity to maximize heat transfer, and have high absorptivity and low emissivity are required to increase the use of CSP. Additionally, these materials must be economically competitive and easy to manufacture. One of the main challenges faced for CSP is to find materials that can meet all these requirements. These include materials with tailored properties or new processing routes that can broaden prospects for CSP in the future^11^.

Metal alloys continue to be key elements in the development of solar receivers. Sarvghad et al.^12^ conducted a comprehensive review of the high-temperature degradation mechanisms of the structural alloys used in CSP. Among these, the 316L stainless steels and the superalloys, both based on Fe and Ni alloys respectively, are particularly noteworthy. Nickel-based superalloys are one of the most widely used materials due to their mechanical strength and oxidation resistance at high temperatures. However, due to the extreme operating conditions not all the superalloys are suitable. For instance, according to Colas et al.^13^, Inconel 625 is not suitable for solar receiver application, because of its poor oxidation resistance at 1373 K in air due to the oxide spallation. Laporte-Azué et al.^14^ investigated the creep-fatigue damage of four alloys commonly used as receivers: Haynes 230, alloy 316H, Inconel 625, 740H, and 800H. The alloys were in the form of seamless tubes. Balat-Pichelin et co-workers^15^ characterized Inconel 617, HR120, Hastelloy X and Haynes 230 superalloys, concluding that the last one presented the best properties to operate at high temperature. In addition to these alloys, Inconel 718 is also a promising alternative due to its combination of mechanical properties, including tensile and creep strength, as well as high temperature resistance^16^. However, optimising the design of solar receivers necessitates not only the identification of novel base alloys but also the development of manufacturing routes capable of facilitating the realisation of more efficient designs. Du et al.^17^ suggested the additive manufacturing as an interesting way to fabricate complex geometries for use in high temperature applications. Furthermore, the impact of heat treatment on the behaviour of AM-fabricated samples should not be overlooked. Recent research by Hu et al.^18^ demonstrates that conventional heat treatments may not be the optimal solution for Inconel 718 produced via additive manufacturing. This finding opens up significant opportunities for future research in this specific AM application.

The aim of this paper is to evaluate the thermal shock resistance of additive manufactured Inconel 718 samples, which could potentially be used as solar receivers. The thermal shock tests were conducted using a vertical continuous solar furnace at the PROMES–CNRS Solar Furnace installation in France.

## Experimental

Inconel 718 superalloy cuboid samples of 20 × 20 × 25 mm^3^ have been produced by Additive Manufacturing (AM) using a Renishaw AM 400 Laser Melting System. This system consists of a high-power 400W Yb-fiber laser interacting simultaneously with the whole powder bed surface to improve build rates. The processing parameters used were 400 W laser power, 1600 mm s^−1^ scan speed, 70 µm laser spot size, 80 µm hatch spacing and 25 µm layer thickness. Renishaw Inconel 718 powder was used, which composition is presented in Table 1.

**Table 1:** Powder composition for preparing additive manufacturing samples*.

Element (wt.%)	Ni	Cr	Fe	Nb	Mo	Ti	Al	Mn	Si
Powder composition*	50–55	17–21	Balance	4.75–5-50	2.80–3.30	0.65–1.15	0.20–0.80	≤ 0.35	≤ 0.35

*According to RenAM500 series material data sheet.

Samples were produced in two different conditions: as built (hereinafter, Sample 1) and thermally treated at 980 °C ± 10 °C for 1 h and air cooled (Sample 2) to relieve internal stresses that result from the manufacturing process.

A die-forged Inconel 718 in a solution-annealed condition was used as a reference material (Reference sample). The solution-annealing treatment consisted of treatment at 980 °C ± 10 °C for 1 h, ageing at 720 °C ± 10 °C for 8 h, and further ageing at 620 °C ± 10 °C for 8 h.

The composition of the samples, both those fabricated by additive manufacturing and the reference material produced by die-forging, was characterised by Energy Dispersive X-ray Fluorescence spectroscopy (EDXRF).

Thermal shock tests were conducted at the Processes, Materials and Solar Energy laboratory (PROMES) of the Centre National de la Recherche Scientifique (CNRS) in Odeillo-Font Romeu (France), using a vertical parabolic solar furnace. It consists of a heliostat primary mirror (located outside the building) and a stationary parabolic secondary mirror (1.5 m diameter). It concentrates solar radiation by a factor of 15,000 on a surface area of 1 cm^2^.The thermal power achieved is up to 2kW. Further details of the facility can be found elsewhere^19,20^.

**Fig. 1:**
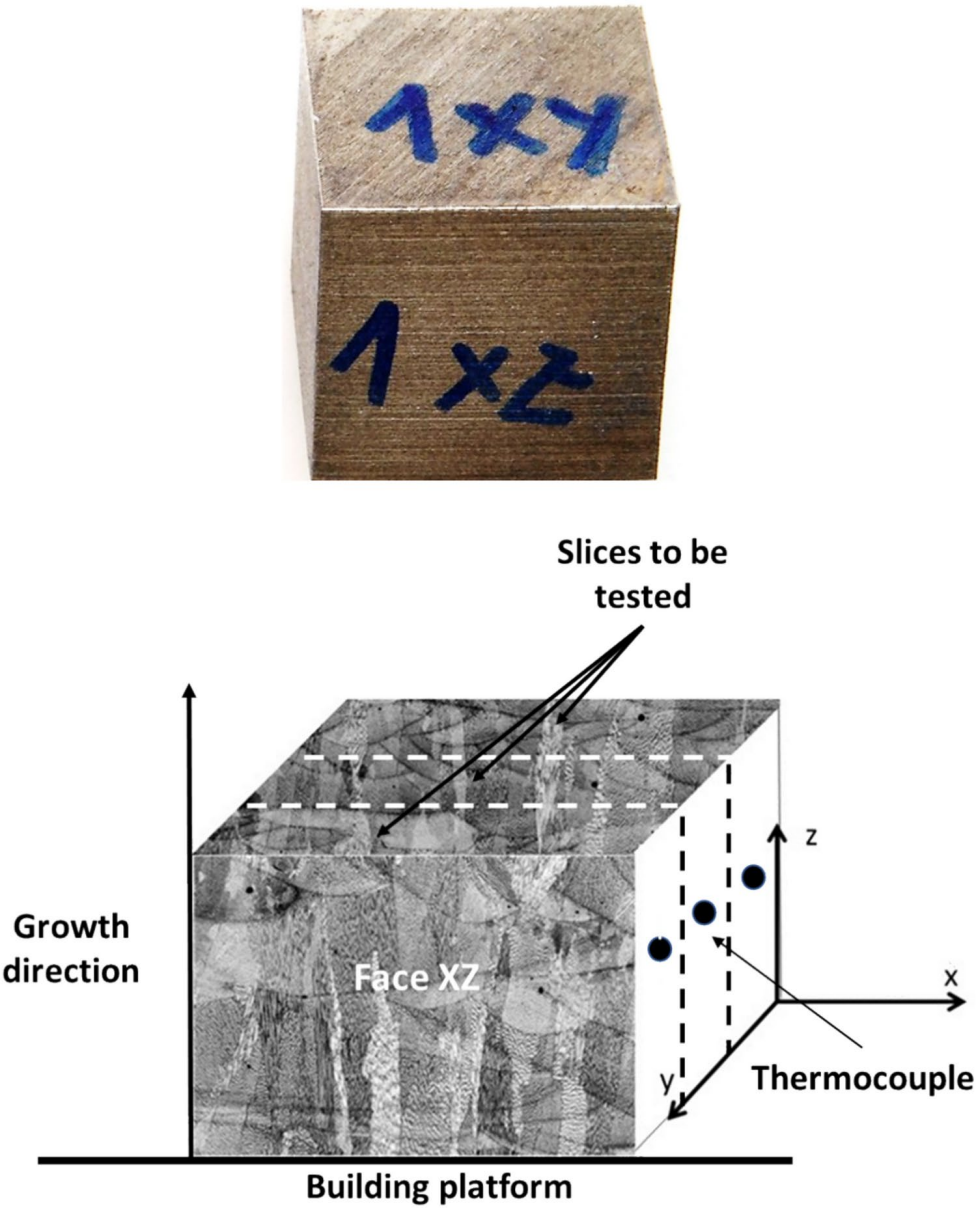
Samples used for thermal shock tests, Up) Solid samples after manufacturing, Down) Scheme of the slices used for testing.

The samples for thermal shock tests were manufactured following the scheme shown in Fig. 1.

**Fig. 2:**
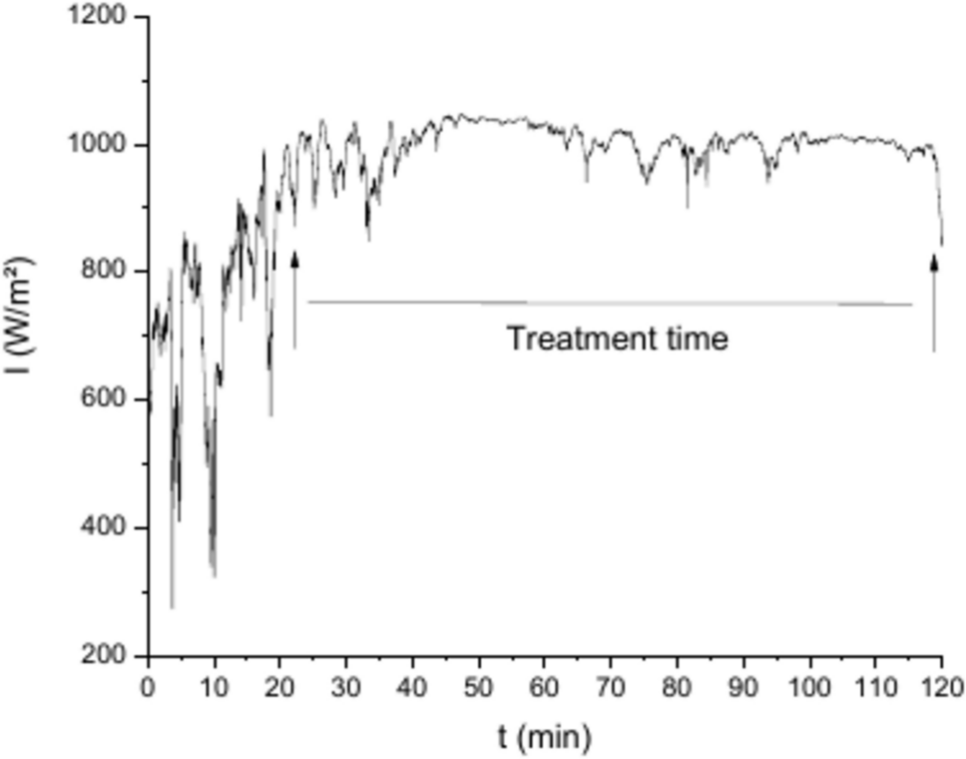
Direct solar radiation fluctuations during a representative thermal shock test. All test were done above 800 W/m^2^.

The samples of 20 × 20 × 7 mm^3^ were positioned at the focus of the solar concentrator, resulting in the heating of the sample. Temperature was recorded continuously throughout the test by a thermocouple placed in a hole (5mm long) in the side of the sample. All experiments were conducted within an irradiance range of 750 to 1060 W/m^2^, despite irradiance changes could happen during the test due to atmospheric conditions (such as passing clouds for instance). These values of irradiance are considerable enough to heat and even melt the sample quickly. Figure 2 shows the direct solar radiation fluctuations during a thermal shock test. All tests were carried out in open air atmosphere.

The temperature on the sample was controlled using a shutter located between the sun-tracking heliostat and the parabolic concentrator, enabling heating and cooling cycles to be carried out. To avoid premature melting, the temperature was gradually increased by progressively opening the shutter. Samples were subjected to ten thermal shock cycles, with an average maximum temperature of 948.98 °C, and an average minimum of 251.02 °C. The heating rate was 14.6 °C/s, while the cooling rate was 4.34 °C/s.

**Fig. 3:**
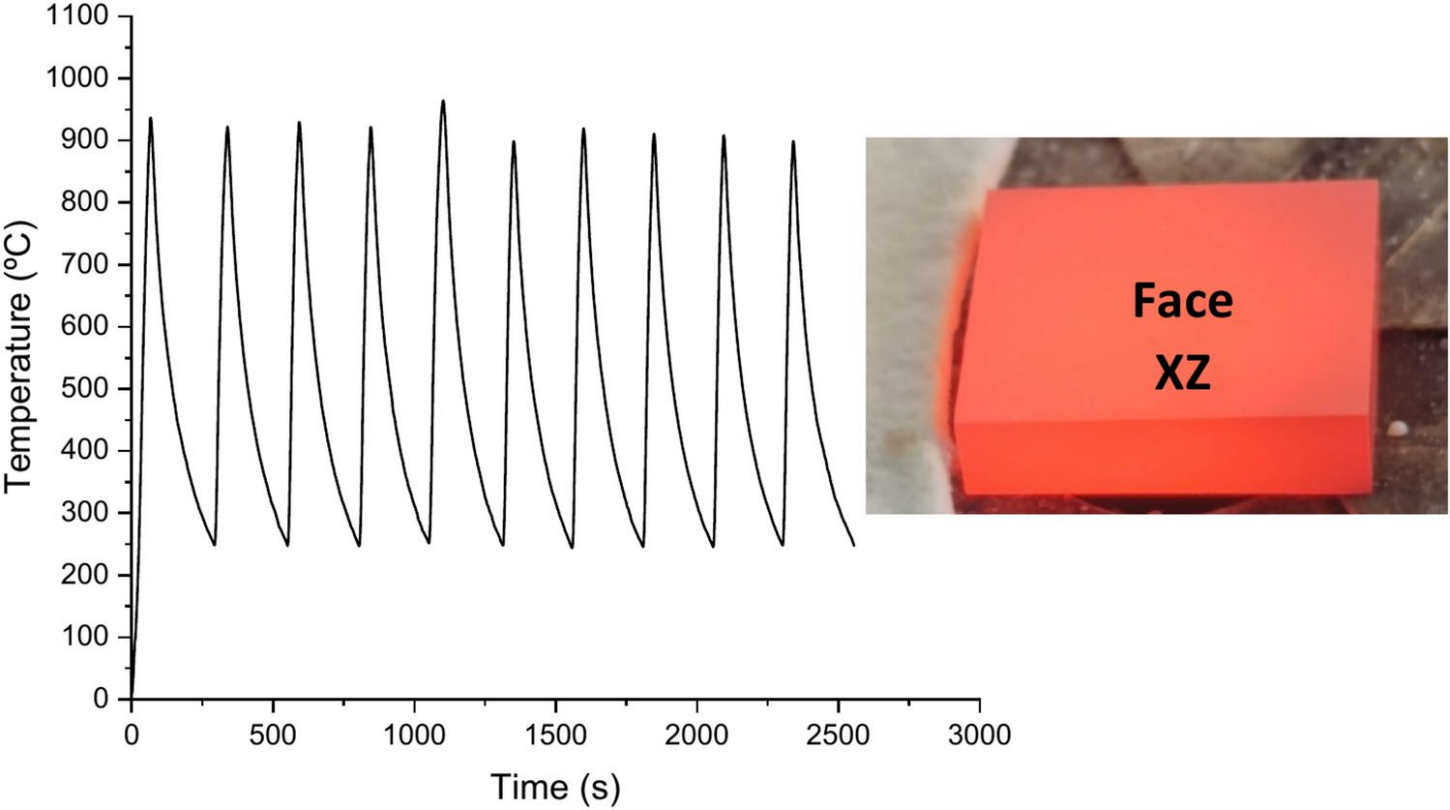
Cyclic temperature profile from 250 to 950 °C as well as the appearance of the sample after 10 cycles.

Figure 3 displays the cyclic temperature profile recorded as well as the appearance of the sample after 10 cycles. The same pattern is repeated for the remaining samples.

After testing, both the surface and the cross-sections of the samples were evaluated using a Hitachi S 4800 J field emission scanning electron microscope (FEG-SEM) with energy dispersive microanalysis (EDS). Inconel samples were etched in a solution of HNO_3_, HCl and H_2_O_2_ or electrolytic etching in 10% oxalic acid solution, 6V, 35 s. The average grain size has been determined using optical microscopy images, followed by analysis with ImageJ software.

X-ray diffraction experiments were performed using a D8 Discover diffractometer (Bruker AXS) equipped with central open Euler ring, horizontal theta-2theta goniometer, Co source (operating at 30 mA, 40 kV), Göbel mirror and Lynxeye linear detector. The working conditions were selected in each case to obtain X-ray diffraction diagrams with good counting statistics and well-defined peaks. The control and data acquisition were automated, using TOPAS 4–2 software to quantify the present phases.

Raman spectroscopy was conducted using a Renishaw Ramascope 2000 Raman spectrometer coupled to an Olympus BH-2 optical microscope. A continuous argon ion laser operating at 514.5 nm (2.4090 eV) was used to excite surface samples. Tests were performed at room temperature with the 100 × microscope objective. Data were collected for 300 s.

## Results and discussion

### Microstructural and compositional analysis

As mentioned previously, the composition of the samples was analysed using X-ray micro fluorescence technique. Table 2 shows the composition of Inconel 718 obtained by both additive and die forging (reference material).

**Table 2:** Elemental analysis determined by Energy Dispersive X-ray Fluorescence spectroscopy (EDXRF).

Sample/element (% mass)	Ni	Cr	Fe	Nb	Mo	Ti	Mn
Reference sample (718 Solution annealed)	53.3	18.5	19.3	4.9	2.8	0.9	0.30
Sample 1 (as built)	53.2	18.8	18.3	5.2	3.4	0.9	0.13
Sample 2 (heat treated)	53.9	18.8	18.5	4.8	2.9	1.0	0.10

As can be seen, all samples have a similar composition regardless of the manufacturing method used (AM or die forging). Slight differences in EDXRF analyses of the same alloy can result from a variety of factors inherent in both the analytical method and the sample characteristics, even with well calibrated instruments which may have inherent statistical variations in the detection of X-ray photons. Relative standard deviations obtained by EDXRF of 1–10% for major elements and 5–25% for minor elements are considered acceptable, which is consistent with the data presented in Table 2.

**Fig. 4:**
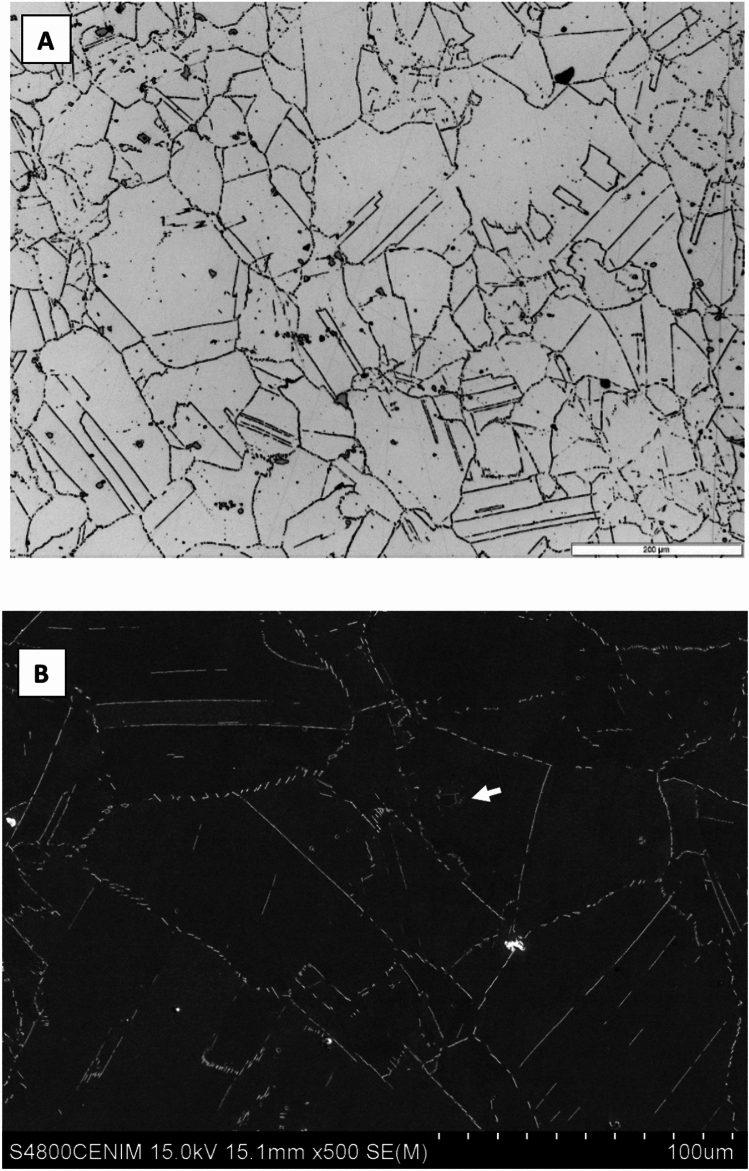
Die-forged solution annealed Inconel 718 microstructure by (**A**) Optical and (**B**) Scanning electron microscopy.

Die-forged solution annealed Inconel 718 presented a microstructure consisting of fine equiaxed grains of γ-austenitic phase, a supersaturated solid solution enriched in Cr and Fe, with transgranular and intergranular second phases as well as MC-type primary carbides, Fig. 4a, possibly Ti and Nb carbides. Upon magnification, fine needles and lamellar particles are clearly observed at grain boundaries and inside grains, as well as polygonal-shaped blocks (see solid arrow in Fig. 4b, etched with oxalic acid).

**Fig. 5:**
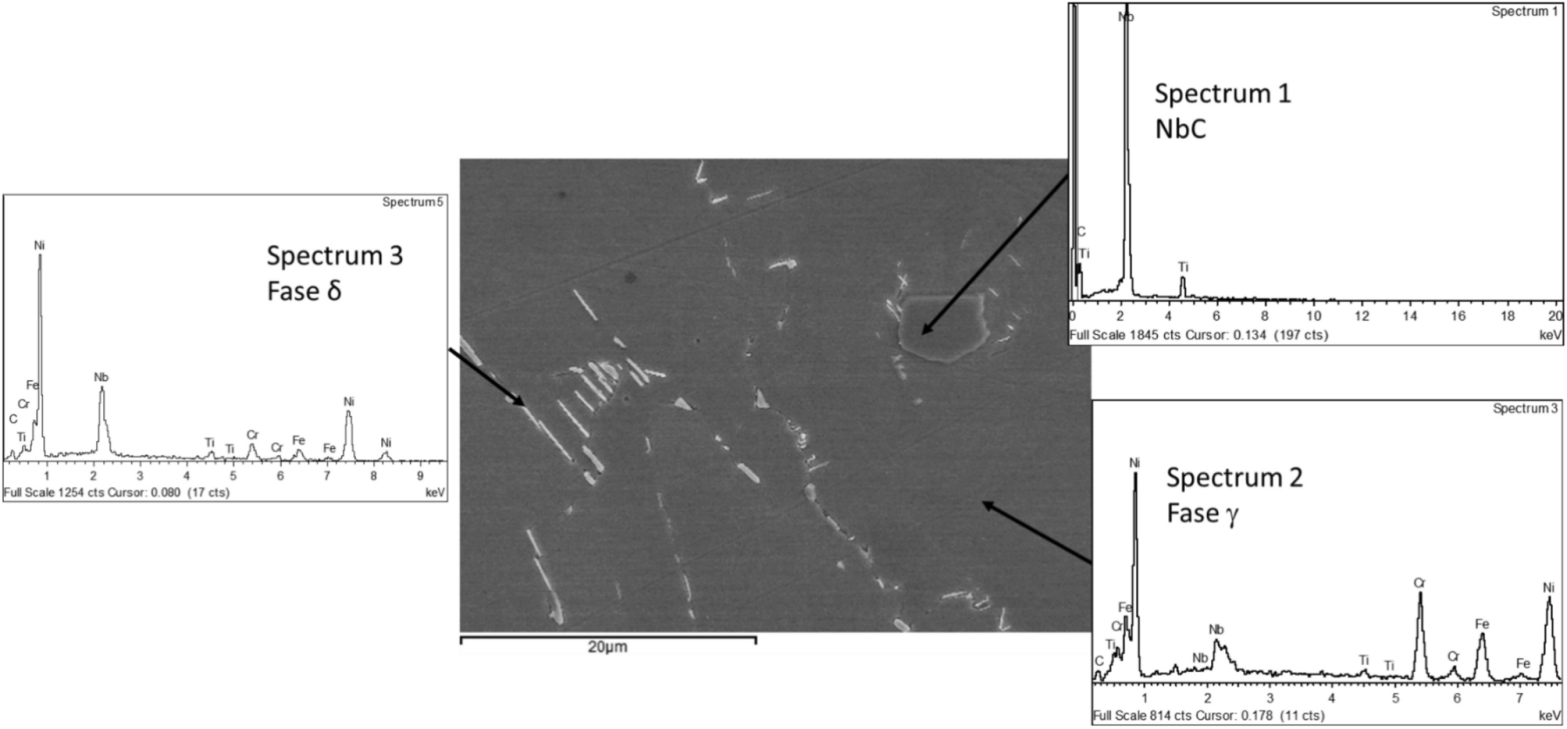
Semi-quantitative analysis using Energy Dispersive Spectroscopy (EDS) with identification of the main constituent phases.

The semi-quantitative analysis performed by Energy Dispersive Spectroscopy (EDS) at different sites in Fig. 5, are showed in Table 3. The composition reveals that these particles are δ phase (Ni_3_Nb) distributed in the austenitic FCC matrix (γ Phase) and NbC and TiC carbides. This microstructure is consistent with those reported in the literature^21^.

**Table 3:** EDS analysis of matrix and particles found in die-forged Inconel 718 in a solution annealed condition (Reference sample).

	C	Ti	Cr	Fe	Ni	Nb
Spectrum 1 (MC-type carbide)	38.65	6.03	–	–	0.00	62.11
Spectrum 2 (γ Phase-matix)	4.42	1.08	18.77	16.73	53.92	5.09
Spectrum 3 (δ Phase-Ni_3_Nb)	8.78	2.21	6.24	5.60	56.25	20.94

**Fig. 6:**
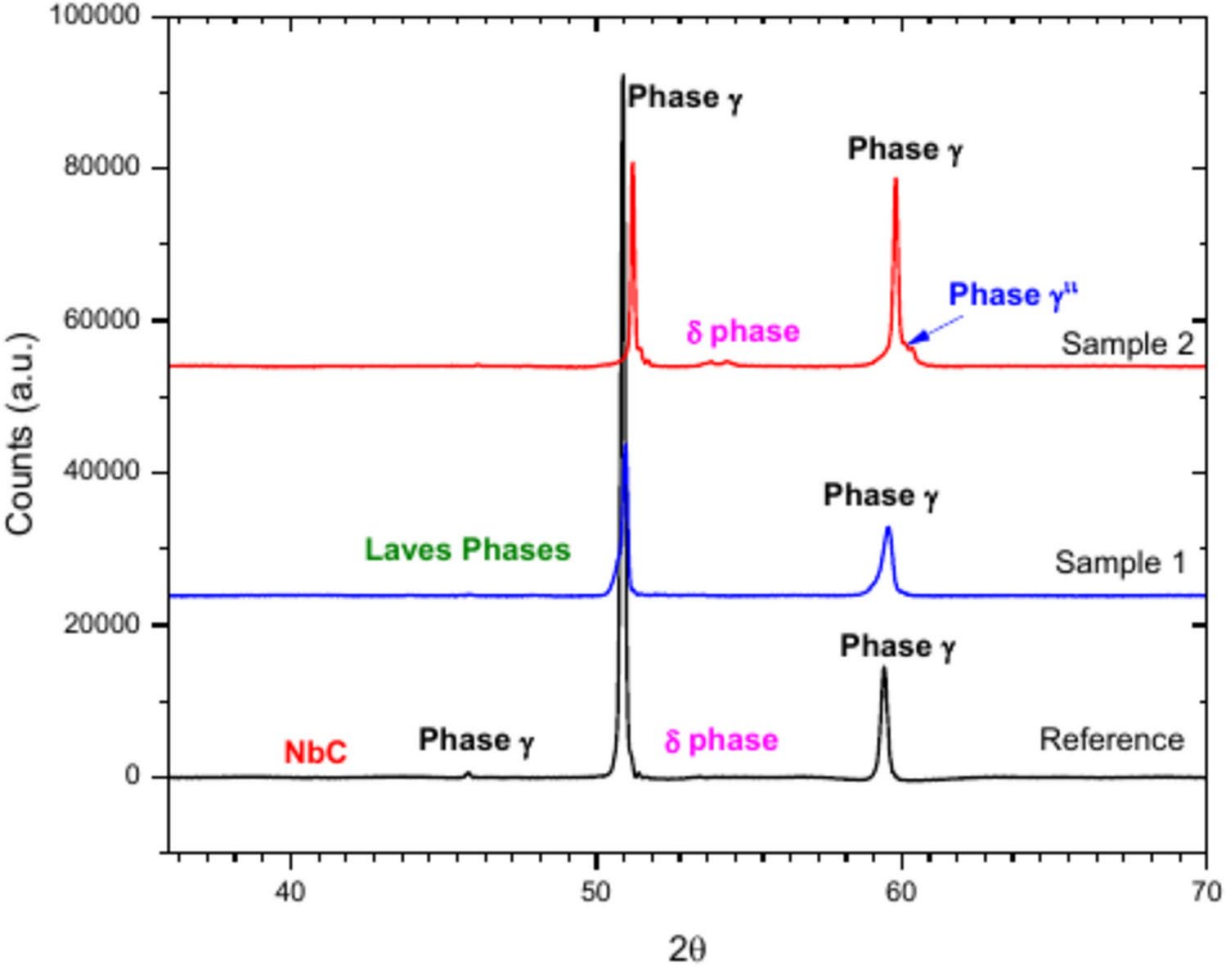
X-ray diffractograms of the three sample surfaces. NbC carbides are also detected by this technique.

X-ray diffractogram depicted in Fig. 6, confirmed the predominance of the austenitic γ phase with a face-centred cubic (fcc) unit cell and NbC carbides. Moreover, the presence of minor quantities of the orthorhombic δ phase, Ni_3_(Nb,Ti), are also observed. In general, the phases present in superalloys depend on both the composition and the thermomechanical treatment. As the number of constituent elements is significant, the phases deviate from their theoretical composition^22^. For this reason, the peaks appear with different intensities and do not fully correspond to those in the theoretical databases. The Rietveld analysis applied to the X-ray diffraction data confirmed the austenitic γ phase as the main phase (98.69%) and smaller fractions of the δ phase (1.19%) and carbides (0.12%). The presence of γ’ and γ” phases would also be possible, but -if present- their content is so low that it cannot be detected either by XRD or SEM observation^23^ because the XRD peaks of both phases (even δ phase) overlap between them. The material has an average grain size of 13 µm.

X-ray diffraction (XRD) analysis of the as-built AM sample (sample 1) revealed that it is primarily composed of γ phase, along with minor intermetallics such as Laves phases. A detailed analysis of the microstructures would require the use of complementary techniques, which were not employed in this study. However, based on the literature, XRD and SEM analyses also suggest the formation of γ and Laves phases. Hu et al.^18^ studied Inconel 718 specimens fabricated by additive manufacturing, found similar results to those find in the present work. Their results revealed the presence of the γ phase and the formation of Laves phases with varying compositions. Their additional transmission electron microscopy (TEM) analysis confirmed the absence of the γ’, γ”, and δ phases.

Conversely, for the heat-treated AM sample (Sample 2), the Laves phases were found to have dissolved, transforming in γ”and δ phases. The presence of the δ-phase -Ni_3_(Nb,Ti)- can be expected to result from the heat treatment described above, Table 4.

**Table 4:** XRD Rietveld quantification of phases presented in the reference sample, sample 1 and sample 2.

Sample	γ Phase	γ” Phase	δ Phase Ni_3_(Nb,Ti)	(Ti,Nb)C	Laves phases
Reference sample (forged 718 )	98.69		1.19	0.12	
Sample 1 (as-built AM)	99.65				0.35
Sample 2 (heat-treated AM)	93.84	3.38	2.85		

**Fig. 7:**
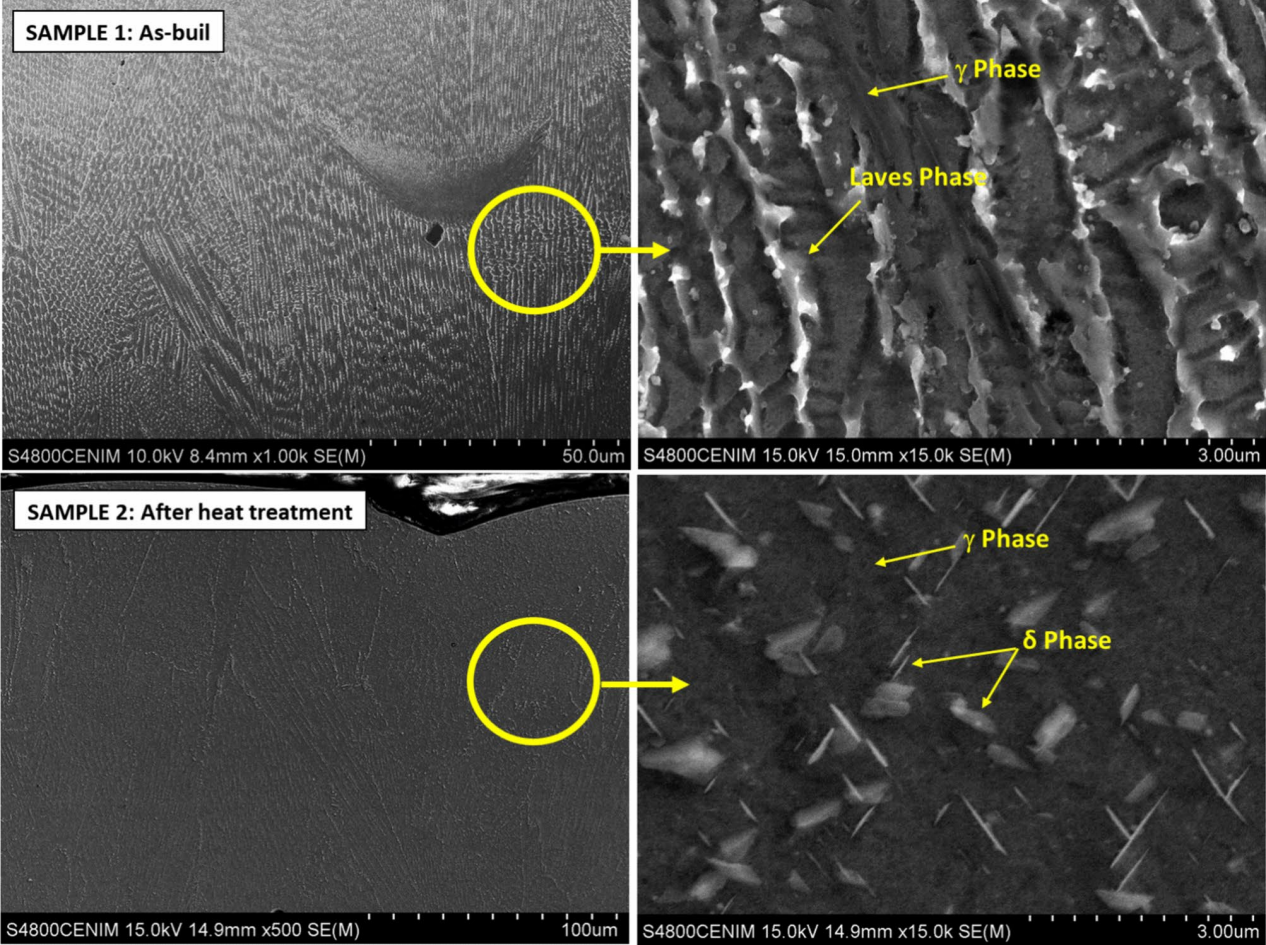
Inconel 718 produced by additive manufacturing in both conditions, as-built (Sample 1) and after heat treatment (Sample 2), showed remarkable differences in microstructure from that produced by forging, Fig. 4.

Inconel 718 produced by additive manufacturing in both conditions, as-built (Sample 1) and after heat treatment (Sample 2), showed remarkable differences in microstructure from that produced by forging (Reference sample), Fig. 7. The microstructure of the manufactured sample, in both the as-built and heat-treated conditions on the XZ plane, clearly reveals a columnar dendritic microstructure aligned parallel to the build axis. This structure exhibits overlapping of the different deposition layers, with evident segregation at the boundaries and the presence of some remaining inclusions. Although the microstructure of the AM samples is logically influenced by the building direction, from a microstructural point of view, there are no major differences among XY, XZ and YZ planes.

Both samples show the chessboard patterns as well as the morphology resulting from the laser melting and the formation of columnar dendrites inside the melt pool. At higher magnifications can be observed that both samples exhibit cellular dendrite microstructure ranging in size from 0.8 to 1.5μm, while the elongated dendrite patterns range in spacing from 1.4 to 2 μm. Huang et al.^24^, using EBSD techniques, described a microstructure composed mainly of coarse columnar grains with fine grains around them, similar to the results shown here.

However, as it is shown in Fig. 7, whereas in as built condition—Sample 1- both the laser tracks and the dendritic structure can be clearly distinguished, in the heat-treated AM sample -Sample 2-, the structure is more homogeneous with less pronounced dendrites.

Moreover, the typical dendritic microstructure of as-built samples (Sample 1) that overlaps more than one melting pool is evident. The grain structure cannot be easily identified in the as-built sample, Fig. 7. The as-built rapid-solidification microstructure disappears after the proper solution heat treatment (followed by standard ageing) that homogenize the material leading to an isotropic microstructure with no preferential growth direction (see micrograph of sample 2). Such a microstructure, with an average grain size of 4 (ASTM E112-18), is consistent with extensive recrystallization of the material. No carbide precipitation can be observed using the analysis tools employed here. Grain texture analysis confirmed that the marked anisotropy of the microstructure between the analysed planes vanishes by a proper solution heat treatment, and the grains misorientation angle analysis confirms a complete recrystallization of the material.

### Thermal shock test

Due to the nature of the present test, which performs 10 cycles between 250 °C and 950 °C in less than 3000 s, determining the growth kinetics of the oxide layers is not feasible. In this respect, additional studies with prolonged exposure times or controlled cycling conditions are required to better understand the oxide layer growth behavior. In this sense, future research could greatly benefit from utilizing the approach known as "unified phase-field modeling (UPFM)," as described by Zhao^25^, to predict oxide layer growth kinetics under cyclic thermal conditions. Focusing on the oxidation kinetics and phase transitions specific to Inconel 718 under thermal shock conditions could help ensure that the UPFM model accurately represents the material’s response to thermal cycling and oxidation. This model serves as an ideal tool, as it comprehensively captures material properties such as thermodynamics, kinetics, composition design, process optimization, microstructure control, and performance prediction, thereby opening new avenues for materials development. Utilizing such a model would enable reliable predictions of the long-term behavior of Inconel 718 under thermal shock conditions, fostering more efficient material utilization and improved design strategies.

**Fig. 8:**
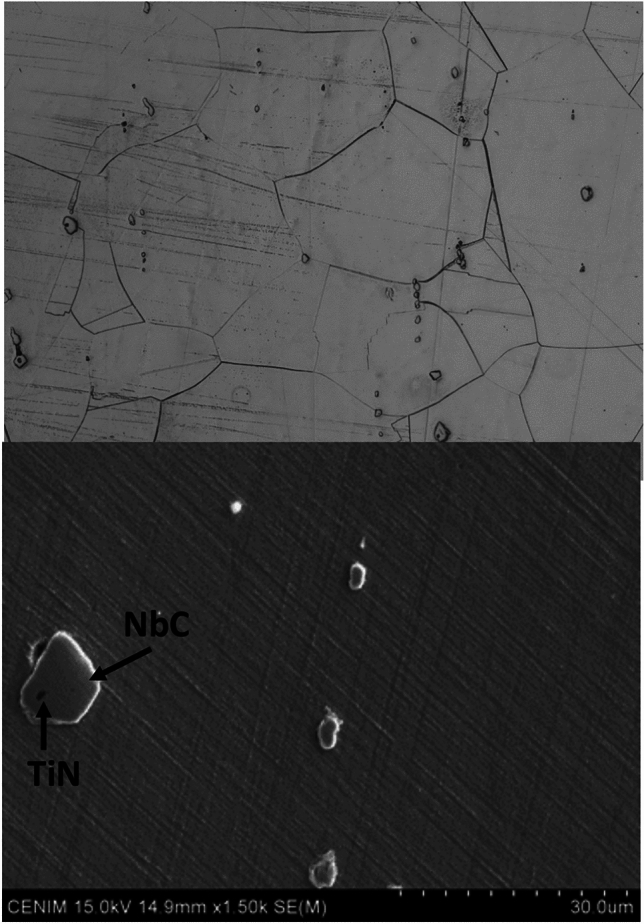
(Top) Microstructure of the reference sample observed by optical microscopy after the thermal shock test (10 cycles); (Bottom) SEM image of the NbC present in the γ matrix.

Firstly, it is important to highlight the microstructural changes observed in each of the materials tested. The microstructure of the reference material consists of austenite with twinned grains, partial recrystallization, and carbides. The grain size has increased substantially to an average of 24 μm. It is interesting to note that the δ phase was not observed; only NbC and TiN were present, as shown in Fig. 8.

**Fig. 9:**
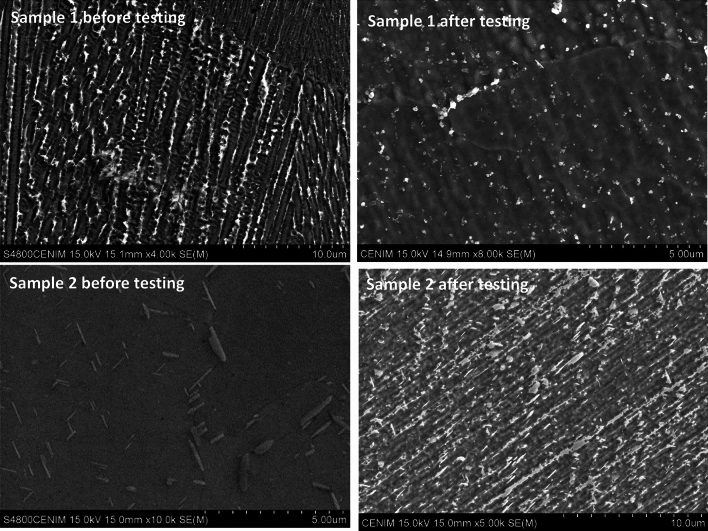
SEM detail of the microstructure of the samples produced by additive manufacturing, before and after testing.

Similarly, the shock thermal treatment acts as a form of homogenisation for the samples obtained by additive manufacturing (AM), significantly reducing micro-segregation and Laves phases due to the diffusion of Nb and Ti into the γ-matrix, Fig. 9.

After the thermal shock test, all samples regardless of the processing route, showed a good visual appearance, with the formation of a continuous black oxide layer on its surface. In general, no spalling of the layer was observed, although the oxide layer is uneven at some points on the edges of the reference sample (forged) and on the as-built AM sample, Sample 1.

**Fig. 10:**
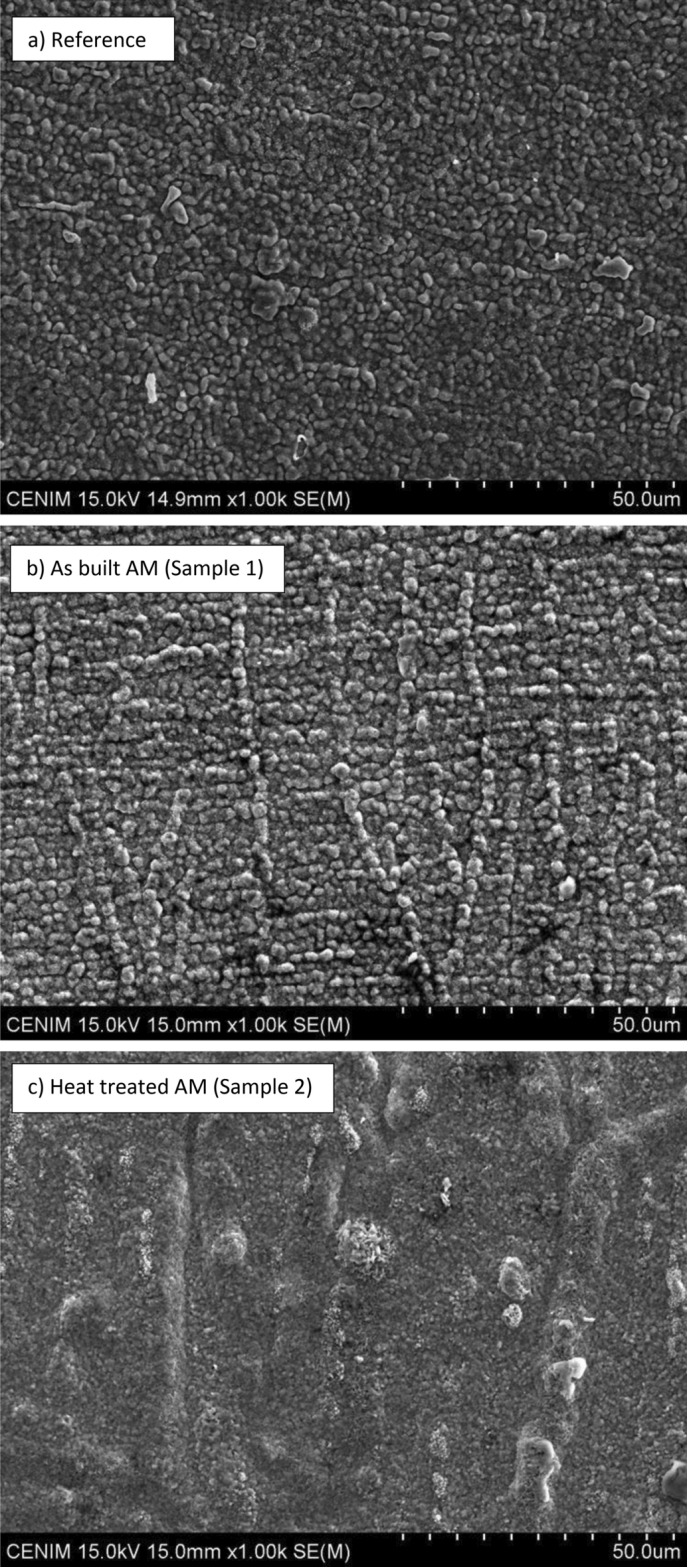
SEM images of the surface morphology of the oxide layer formed on the surface of each tested material after the cyclic thermal shock test between 250 and 950 °C.

Figure 10 shows the SEM images of the surface morphology of the oxide layer developed on grounded surfaces after the cyclic thermal shock test between 250 and 950°C. All samples show a uniform oxide scale over the entire surface, as shown in same figure.

However, slight variations in morphology were observed between the samples depending on whether they were forged or additive manufactured and the type of heat treatment applied. While Sample 2—heat treated AM samples- appears to be more compact (Fig. 10c), the reference sample (die-forging) and Sample 1 -as-built AM sample- have a coarser structure of swelling ridges, Fig. 10a, b, respectively. These features are formed as micro-pillars in the forged material (reference sample), whereas in Sample 1 they resemble a "worm’s trail”.

**Fig. 11:**
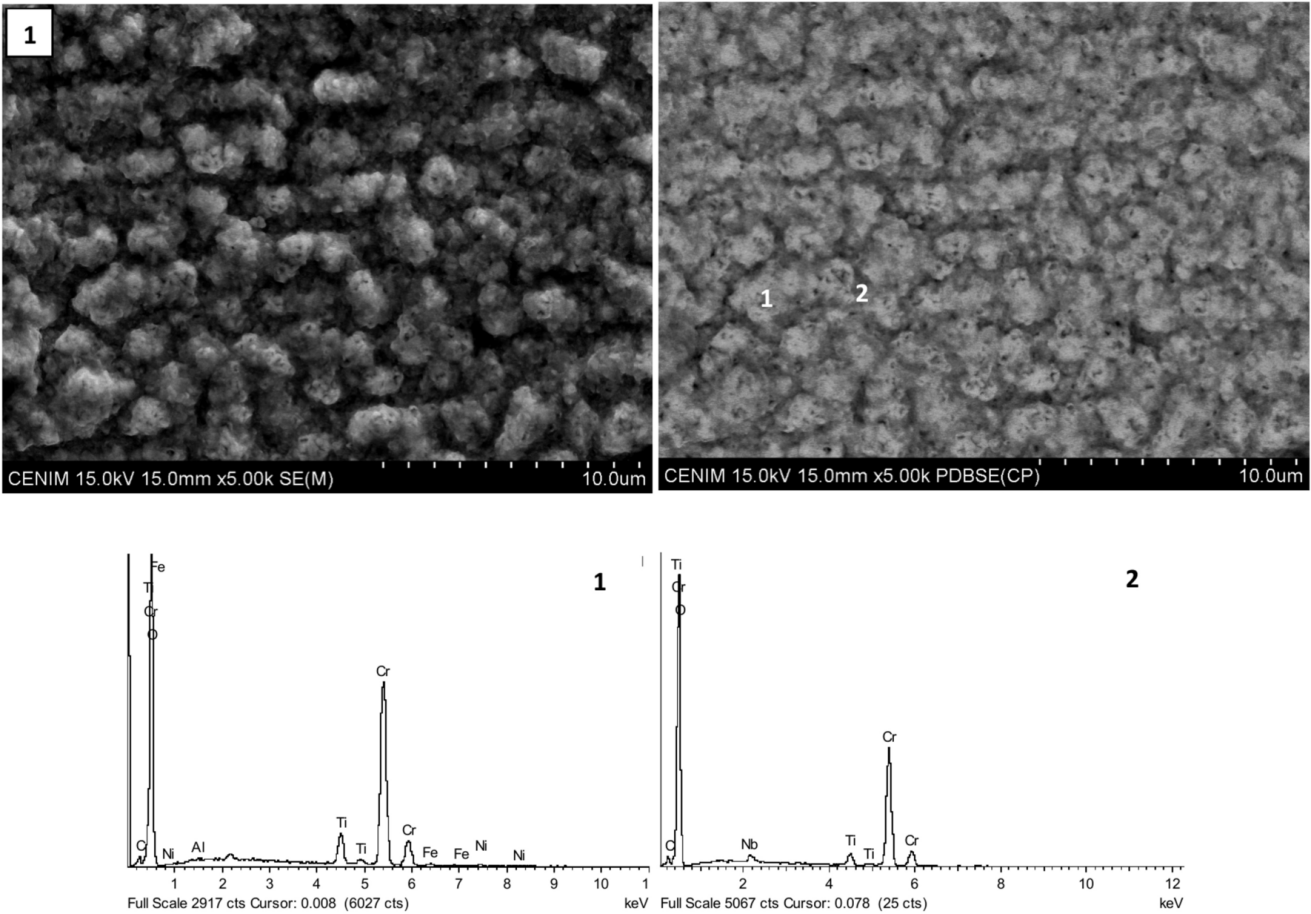
An enlarged view of the surface corresponding to the as-built AM sample 1.

Figure 11 shows a close-up view of the surface corresponding to the as-built AM -Sample 1. EDS analysis confirmed the presence of chromium and oxygen at the surface and Nb in the deeper zones of the surface.

**Fig. 12:**
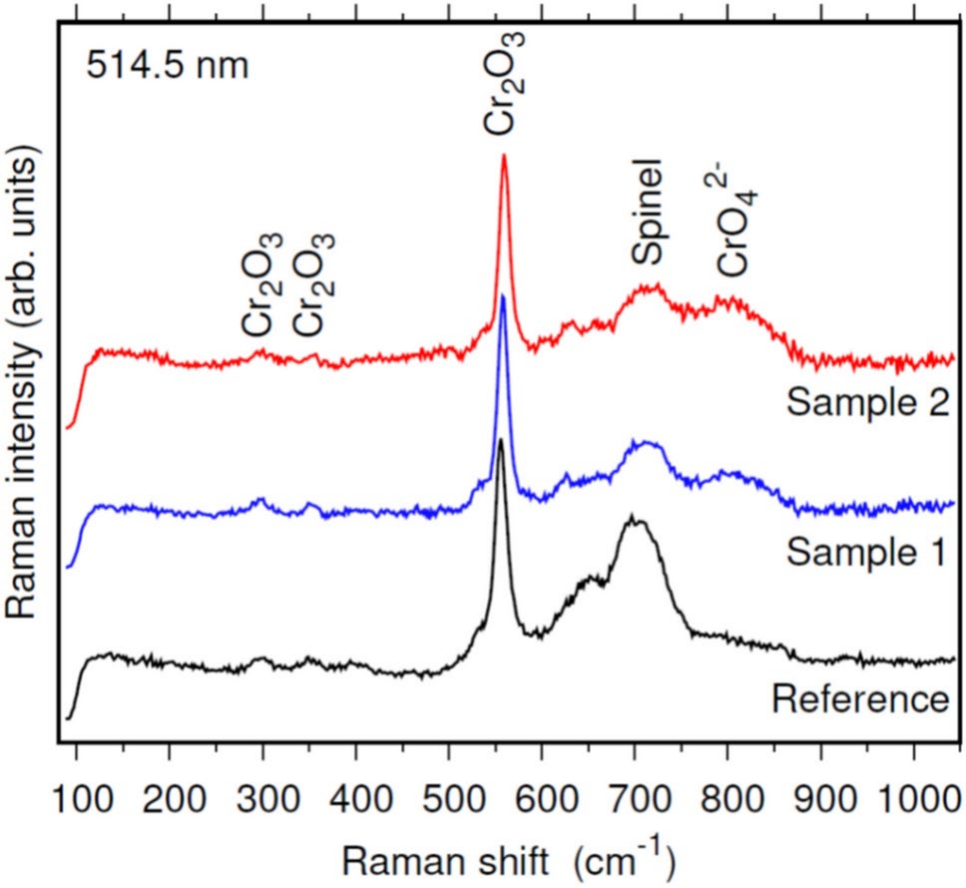
Raman spectra of the oxidised samples, including the reference and AM-produced samples.

This composition agrees with the results obtained by Raman spectroscopy. Figure 12 displays the Raman spectra of the oxidised samples, including the reference sample and the samples produced by AM process. As it can be seen the spectra are quite similar, comprised by a main peak at 560 cm^-1^ associated to Cr_2_O_3_^26–28^; the Ni(Cr, Fe)_2_O_4_ spinel phases are also observed at 695–710 cm^−1^ ^29^ and, finally, a weak peak related to CrO_4_^2−^ ions is observed at 820 cm^-1^. The spinel phases appear to increase relative to Cr_2_O_3_ and CrO_4_^2−^ ions for the reference sample (forged). The small penetration depth of the Raman spectroscopy suggests that the outermost surface of the oxide layer is mainly composed of crystalline Cr_2_O_3_ for all samples.

**Fig. 13:**
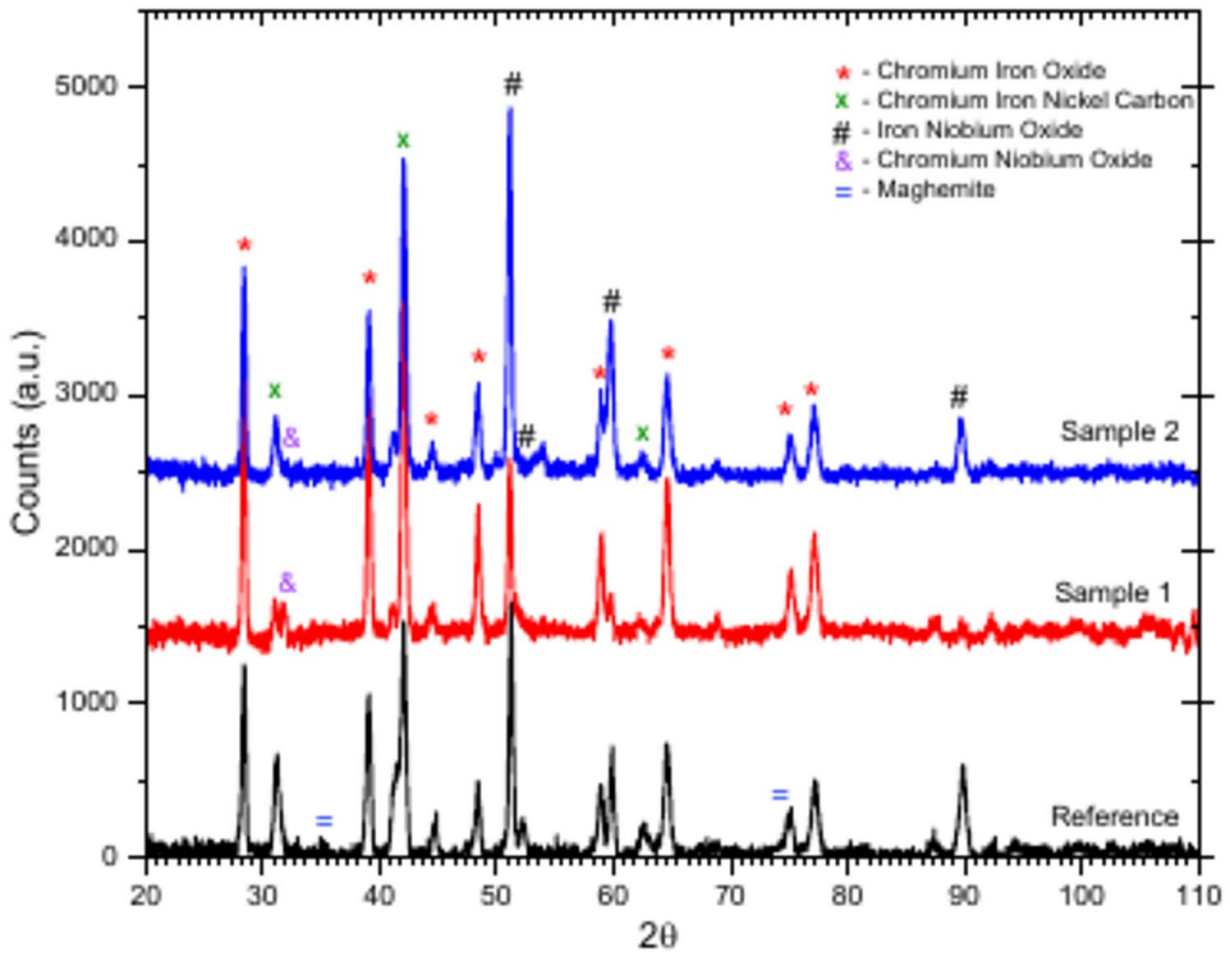
Grazing Incidence X-ray Diffraction (GIXRD) characterisation of oxidised surfaces with peaks corresponding to the phases present in the oxide layer.

Grazing Incidence X-ray Diffraction (GIXRD) performed on the surface of oxidized samples, Fig. 13, provides complete information of the phases present at the oxide layer. GXRD diffractograms reveal the formation of chromium iron oxide (Cr_1.3_Fe_0.7_O_3_), chromium nickel carbon (FeCr_0.29_Ni_0.16_C_0.06_), iron niobium oxide (FeNbO_4_) and chromium niobium oxide (CrNbO_4_). In addition, the reference sample produced by die-forging also showed the presence of maghemite, a spinel structure (AB_2_O_4_) similar to magnetite (Fe_3_O_4_)^30^. This could be related to the less adherent protective layers formed on its surface during the thermal shock tests. Doleker et al.^31^ investigated the oxidation of the 718 alloy in an open-air atmosphere at 1000°C for 8, 24, and 100 h. Despite the different experimental conditions of this work, the trends observed were remarkably similar. These authors reported an oxide layer of 4.4 μm in thickness after 8 h, composed by NiCr_2_O_4_ spinel, CrNbO_4_, Ni_3_Al, TiNb_2_O_7_, and mainly chromium oxide Cr_2_O_3_.

**Fig. 14:**
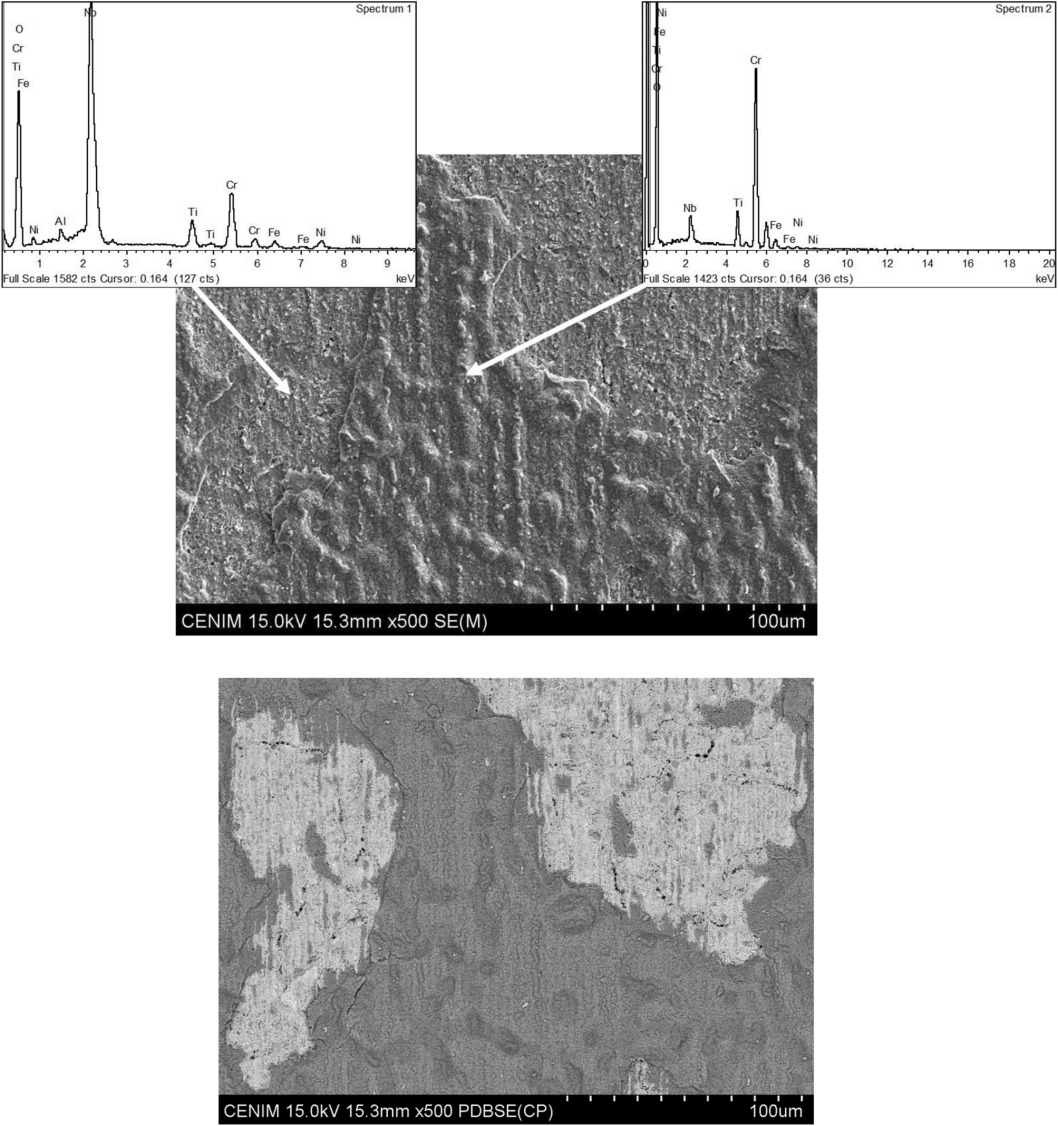
Minor spalling of the oxide layer on the reference sample and the as-built AM sample 1 with corresponding EDS analysis on these regions.

As mentioned previously, at the edges minor spalling of the oxide layer was observed for the reference sample (forged) and the as-built AM processed sample, Sample 1, as shown in Fig. 14. In this figure, EDS analysis shows the presence of niobium and oxygen in the spalling areas, suggesting that there is an inner Nb-enriched oxide layer at the oxide/substrate interphase. Literature^32^ found that 718 specimens produced through additive manufacturing under comparable conditions to those tested here (as-built and lacking heat treatment) had an external layer of chromium spinel (with other compounds), and a thin inner layer enriched in niobium, which appears to behave as a diffusion barrier.

The absence of spalling observed in Sample 2, heat -treated AM, can be explained by the heat treatment applied after sample processing. According to Sanviemvongsak et al.^33^ the homogenization of the microstructure has a positive effect on adhesion of the oxide layer to the surface.

**Fig. 15:**
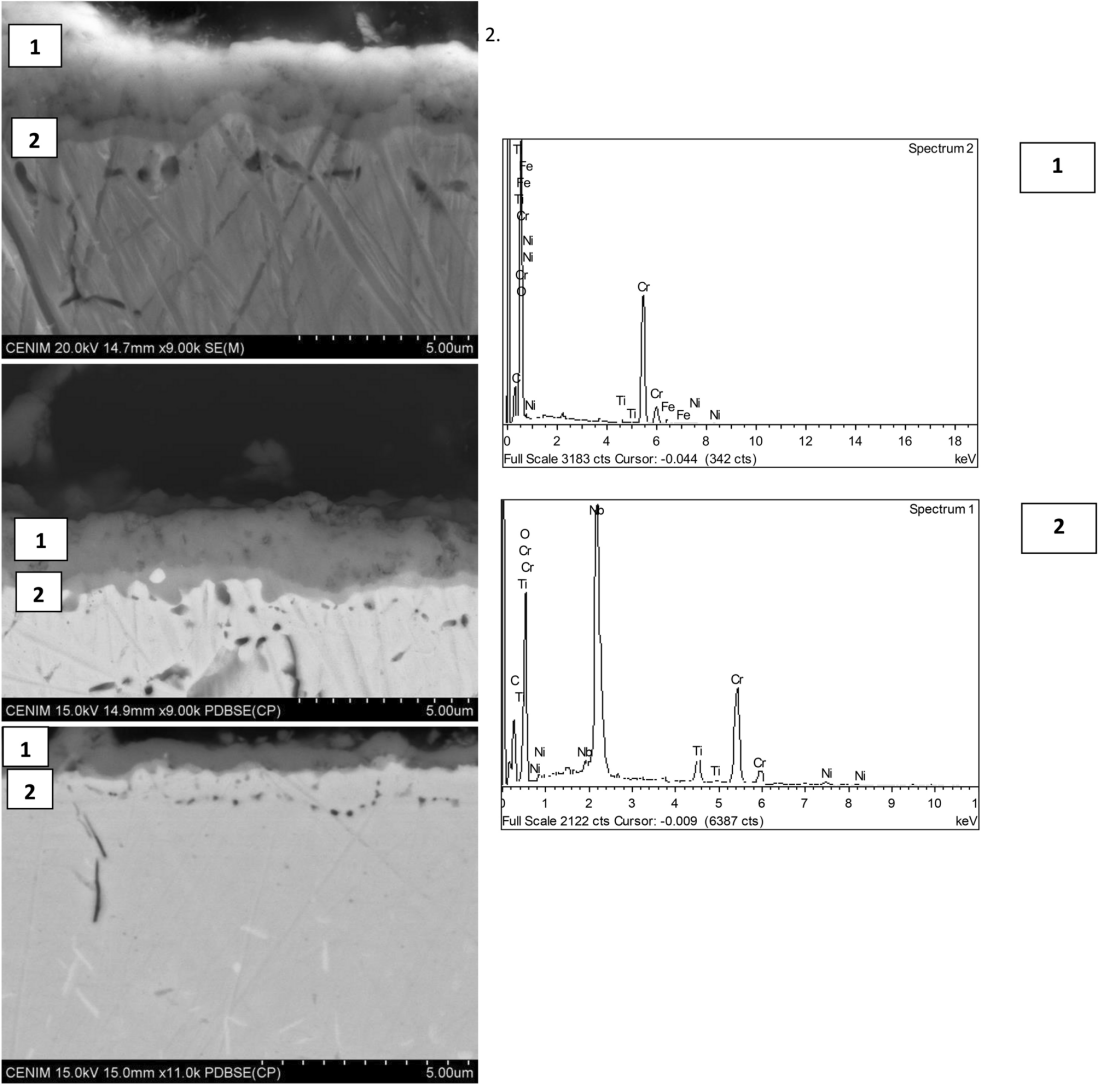
SEM cross-sectional analysis of samples after 10 thermal shock cycles. An outer layer enriched in chromium oxide and an inner layer enriched in niobium oxide can be seen.

Cross-sectional analysis by SEM of the samples after 10 thermal-shock cycles confirms that the oxide layer formed is actually a duplex oxide layer, Fig. 15, with a chromium oxide enriched outer layer and niobium oxide enriched inner layer.

The thickness of the oxide layer is also different depending on the sample. The reference sample had a thickness of 2.5 μm for the outer layer and 0.5 μm for the inner layer enriched in niobium oxide. The additive manufactured samples had significantly lower values for both layers, with Sample 1 measuring 1.80 μm and 0.4 μm for the outer and inner layers respectively, and Sample 2 measuring 0.5 μm and 0.2 μm, respectively. The differences observed in the microstructure of the samples might also explain the variation in the thickness of the oxide. It appears that the forged sample, with a coarser grain size, exhibits a significantly larger growth, followed by the as-built AM whose dendritic structure was more clearly resolved than in the HT-AM.

Table 5 presents the average surface hardness and surface roughness for each of the materials tested. Although the hardness values cannot be considered statistically significant due to the dispersion of results, they can still be regarded as indicative. According to these results, sample 2 exhibited the highest surface hardness and the lowest roughness. This is consistent with its better performance against thermal shock, likely due to the thinner, more compact, and homogeneous layers.

**Table 5:** Surface hardness and roughness in the reference sample, sample 1 and sample 2 after shock thermal testing.

Sample	Hardness (HV0.5)	Roughness (Ra, μm)
Reference sample (Die-forged 718 )	325 ± 2.03	0.218 ± 0.03
Sample 1	369 ± 1.23	0.159 ± 0.023
Sample 2	392 ± 4.05	0.108 ± 0.08

The growth of the oxide layer is a consequence of the diffusion of oxygen and chromium through the lattice and the grain boundary. The diffusion rate at the grain boundary is faster than that of the lattice, resulting in the thickest oxide layer in the reference Inconel 718, which has a microstructure of equiaxed grains. Chen et al.^34^ studied de microstructure refinement of Inconel 718 by adding TiO_2_ nanometre particles and observed that with the decrease of the grain size, the thickness of the oxide layer decreases and also is more compact, providing better oxidation resistance. Moreover, Li et al.^35^ studied influence of the building direction of Inconel 718 superalloy fabricated by electron beam melting, on the oxidation behaviour. These authors reached the conclusion that the boundaries are the preferred sites for oxidation, and that the oxidation behaviour is significantly affected by the elements’ diffusion. It can thus be concluded that the optimal oxidation behaviour is achieved for the building direction that leads to the lowest grain boundary length density, resulting in the formation of a continuous, crack-free oxide layer. Ju et al.^36^ explains, the thickening of the chromium oxide layer prevents the outward diffusion of Nb, as well as other elements such as Ti, resulting in the formation of the inner Nb-rich layer. As the surface oxidizes, intermetallic compounds δ-Ni_3_Nb in the matrix incorporate Nb to form an inner oxide layer. Wang et al. investigated the effects of thermal shock between 960 °C and room temperature on the microstructure and tensile properties of the IN718 alloy after three distinct heat treatments. They observed the formation of protective and dense oxide films on the alloy’s surface, primarily composed of Cr₂O₃, Ti₀.₉₅Nb₀.₉₅O₄, and (Cr, Fe)₂O₃, findings consistent with our results. Furthermore, they reported that thermal shock altered the distribution and quantity of δ phases, a phenomenon also observed in this study.

Experimental results are essential to inform the physical models used to simulate the behavior of Inconel 718 under oxidation and thermal shock conditions. These models aim to predict material performance, facilitate optimal material selection, and support intelligent material design. These results highlight the potential of concentrated solar energy for studying the behaviour of metallic materials under extreme service conditions. Achieving heating and cooling rates like those obtained with this method is challenging using other techniques, which opens up new opportunities for its application in fields such as gas turbines and jet engines. Additionally, additive manufacturing (AM) can be leveraged to influence specific designs, leading to material savings, increased efficiency, and extended service life.

## Conclusions

Concentrated solar energy can play an important role in the testing of metallic materials. By focusing the solar beam, the high temperatures required to change the properties of metals are achieved, allowing the suitability, performance and durability of new materials developed for solar receivers to be tested. Additionally, it offers a sustainable alternative to other methods, reducing the environmental impact of metal testing.

In the present work, a vertical parabolic solar furnace was employed to perform ten heating/cooling cycles between 250 and 950 °C in approximately 250 s per cycle. This is a markedly faster process than that achievable through traditional methodologies.

After the thermal shock tests, all specimens exhibited the formation of a protective oxide layer on their surface. However, the layer that formed on Sample 2 (fabricated by additive manufacturing (AM) and subsequent heat treatment), appears to be more compact and adherent than that formed on the reference material (forged sample). In the last case, the oxide layer presented minor points of spalling on the edges of the sample likely due to its microstructure. The forged material consists of coarse grains, where the boundaries act as diffusion paths for both inward oxygen and outward alloying elements. In contrast, samples produced by AM and heat-treated exhibit a fine dendritic microstructure, resulting in the formation of a more evenly continuous and thinner oxide layer.

## Data Availability

All data generated or analysed during this study are included in this published article.
